# Differences in Youth and Adult Physical Activity in Park Settings by Sex and Race/Ethnicity

**DOI:** 10.5888/pcd10.120276

**Published:** 2013-03-28

**Authors:** Andrew T. Kaczynski, Sonja A. Wilhelm Stanis, Gina M. Besenyi, Stephanie Child

**Affiliations:** Author Affiliations: Sonja A. Wilhelm Stanis, University of Missouri, Columbia, Missouri; Gina M. Besenyi, Stephanie Child, University of South Carolina, Columbia, South Carolina.

## Abstract

We examined differences by sex and race/ethnicity in the observed moderate- to vigorous-intensity physical activity (MVPA) of youth and adults in diverse areas of 4 parks in Kansas City, Missouri, in 2009. Male youth were more active on playgrounds and pools or splashpads than female youth. White youth were less active than nonwhite youth in open spaces and on paved trails. Male adults were more active in open spaces than female adults, and white adults were more active on paved trails than nonwhite adults. Understanding variations in MVPA between user groups can inform park design efforts to foster increased activity among all visitors.

## Objective

Use of parks has potential for lowering levels of physical inactivity and obesity in the United States (1,2). Previous research has demonstrated variations in park-based moderate- to vigorous-intensity physical activity (MVPA) by sex, race/ethnicity, and age (3,4), but few studies have explored users’ MVPA levels by park activity area or how the levels vary by sociodemographic characteristics. Analyzing park use by these characteristics can improve understanding of how park features exacerbate or attenuate disparities in health-promoting MVPA. The purpose of this study was to examine differences by sex and race/ethnicity in the MVPA of youth and adults observed in various park settings.

## Methods

We conducted this study during July and August of 2009 in 4 parks in Kansas City, Missouri (26–129 acres). According to 2000 census data, the median annual household income of the tracts in which each park’s centroid was located ranged from $34,095 to $229,333, and the percentage minority population ranged from 4% to 46% (5). Each park was subdivided into 14 to 28 observable target areas on the basis of natural activity zones or sightline barriers. Further details about the study, which was approved by the institutional review board at Kansas State University, have been reported previously (4).

The System for Observing Play and Recreation in Communities was used to document the MVPA behavior of park users (6). Sex was recorded as male or female; age group as child (aged 2–12 y), teenager (aged 13–20 y), adult (aged 21–59 y), or senior (aged ≥60); race/ethnicity as white, black, Asian, Hispanic, or other/unsure; and MVPA intensity level as sedentary, moderate, or vigorous (6). Each park was observed for 39 hours (Friday through Sunday, 7:00 am to 8:00 pm), which were spread across 2 weekends per park.

Separate analyses are reported for “youth” (child and teenage groups) and “adults” (adult and senior groups). To ensure a sufficient sample size to make racial/ethnic comparisons, Asian, black, and Hispanic users were collapsed into a nonwhite category. Of 13 unique activity areas observed in the study, the 5 areas that were most used by youth and adults were identified. We used logistic regression to examine the likelihood of engaging in MVPA in each park area by male versus female users and white versus nonwhite users. Significance was set at *P* < .05.

## Results

We observed 2,454 youth during the study; 50.2% (n = 1,233) were female, 50.2% (n = 1,231) were white, and 54.3% (n = 1,332) were engaged in MVPA. The most popular areas for youth were trails (n = 678), playgrounds (n = 651), open spaces (n = 504), pools/splashpads (n = 258), and picnic shelters (n = 201). Overall, male youth were more likely to be observed to be engaged in MVPA than female youth (odds ratio [OR] = 1.36; 95% confidence interval [CI], 1.16–1.60), but no significant difference was observed between white and nonwhite youth (OR = 0.89; 95% CI, 0.76–1.05) (Table 1). Male youth were more likely to be observed being active than female youth on playgrounds (OR = 1.62; 95% CI, 1.17–2.24) and pools/splashpads (OR = 2.33; 95% CI, 1.42–3.84). White youth were less likely to be observed being active than nonwhite youth in open spaces (OR = 0.68; 95% CI, 0.47–0.99) and on paved trails (OR = 0.53; 95% CI, 0.38–0.73).

**Table 1:** Youth MVPA, by Sex and Race/Ethnicity, in Park Activity Areas, Kansas City, Missouri, 2009^a^

User Characteristic	Odds Ratio (95% Confidence Interval)					
	All Areas	Paved Trail	Playground	Open Space	Pool/Splash Pad	Picnic Shelter
Female	1 [Reference]					
Male	1.36 (1.16–1.60)^b^	1.11 (0.82–1.52)	1.62 (1.17–2.24)^b^	1.33 (0.94–1.89)	2.33 (1.42–3.84)^b^	1.15 (0.64–2.07)
Nonwhite	1 [Reference]					
White	0.89 (0.76–1.05)	0.53 (0.38–0.73)^b^	1.11 (0.80–1.54)	0.68 (0.47–0.99)^c^	0.73 (0.43–1.22)	1.71 (0.84–3.51)

Abbreviation: MVPA, moderate- to vigorous-intensity physical activity.

a Data collected from Kansas City, Missouri, parks, July and August of 2009. All analyses controlled for sex, race/ethnicity, and the park where the observation occurred.

b *P* < .01.

c *P* < .05.

A total of 6,401 adults were observed; 51.5% (n = 3,297) were female, 68.5% (n = 4,383) were white, and 44.6% (n = 2,854) were engaged in MVPA. Areas most used by adults were paved trails (n = 2,770), open spaces (n = 1,412), playgrounds (n = 531), picnic shelters (n = 464), and tennis courts (n = 336). Overall, male adults were not more likely to be engaged in MVPA than female adults (OR = 1.07; 95% CI, 0.97–1.18), but whites were more often observed being active than nonwhites (OR = 1.32; 95% CI, 1.19–1.48) (Table 2). Male adults were more likely to be active than female adults in open spaces (OR = 1.30; 95% CI, 1.02–1.66). Compared with nonwhite adults, white adults were more likely to be active on paved trails (OR = 1.20; 95% CI, 1.01–1.45) but less likely to be active at picnic shelters (OR = 0.36; 95% CI, 0.21–0.62).

**Table 2:** Adult MVPA, by Sex and Race/Ethnicity, in Park Activity Areas, Kansas City, Missouri, 2009^a^

User Characteristic	Odds Ratio (95% Confidence Interval)					
	All Areas	Paved Trail	Open Space	Playground	Picnic Shelter	Tennis Court
Female	1 [Reference]					
Male	1.07 (0.97–1.18)	0.99 (0.84–1.16)	1.30 (1.02–1.66)^b^	1.14 (0.75–1.74)	1.52 (0.90–2.59)	1.06 (0.63–1.77)
Nonwhite	1 [Reference]					
White	1.32 (1.19–1.48)^c^	1.20 (1.01–1.45)^b^	0.91 (0.71–1.17)	1.19 (0.78–1.83)	0.36 (0.21–0.62)^c^	0.81 (0.51–1.30)

Abbreviation: MVPA, moderate- to vigorous-intensity physical activity.

a Data collected from Kansas City, Missouri, parks, July and August of 2009. All analyses controlled for sex, race/ethnicity, and the park where the observation occurred.

b *P* < .05.

c *P* < .01.

## Discussion

Our findings are consistent with literature showing that boys are more active than girls overall and that no significant variations exist in youth by race/ethnicity (7). Among adults, we found no disparity by sex in MVPA levels overall, but, like other researchers, we found that whites were more likely to be engaged in park MVPA than minorities (8). Our findings suggest that differences in the way users engage in MVPA at parks may be attributable to areas in the park environment, and they have implications for how the design of park settings may help to reinforce or, preferably, mitigate disparities in MVPA among female and minority populations.

We found that adult women were less likely than men to be observed in MVPA in undeveloped open spaces, which may be the result of the unstructured nature of these areas. A recent study found that female park users accumulated more physical activity in parks that offered structured environments (ie, recreation classes) (9). We also found that female youth were less likely than male youth to be active on playgrounds. Explanations for this disparity might include safety conditions regarding the playground facility (10) and sex differences in activity preferences (7). Park planners and programmers may take these preferences for structure, social affiliation, or safety into account in designing park spaces to promote greater MVPA among younger and older women.

We also observed differences by racial/ethnic groups, including greater MVPA among nonwhite youth than white youth in open spaces and among nonwhite adults than white adults at picnic shelters. Previous research has found that nonwhite groups report using parks to spend time as a family or in social groups (8). Therefore, parks with gathering areas such as picnic shelters and adjacent open spaces may serve as a resource for nonwhite youth and family members to engage in outdoor play. Conversely, white adults were more active on paved trails than nonwhite adults, a finding that supports previous findings (11), but nonwhite youth were more active than their white counterparts on paved trails. Paved trails are frequently cited as important MVPA resources in parks, given their multifunctional nature (11), and future initiatives should explore how nonwhite youth’s propensity for active paved trail use can be translated into adulthood.

This study was limited by the need to aggregate all nonwhite race/ethnicity groups, the small number of parks examined, and the comparisons drawn for only the 5 park target areas most used by youth and adults. Nevertheless, our findings support the notion that race/ethnicity, sex, and preference for activity types differentially contribute to park-based MVPA for adults and youth. Future research should explore variations between visitors from diverse racial/ethnic minority backgrounds and why park users from different groups accrue unequal amounts of MVPA in various areas, including purpose of park visitation, choice of activity area, and social/environmental interactions that support park-based MVPA. A better understanding of how and why certain groups use public parks may ultimately lead to an increase in MVPA for all users.
